# Up-regulated expression of oxytocin mRNA in peripheral blood lymphocytes from first-episode schizophrenia patients

**DOI:** 10.18632/oncotarget.20252

**Published:** 2017-08-14

**Authors:** Xiudeng Yang, Yamei Tang, Qinling Wei, Bing Lang, Huai Tao, Xianghui Zhang, Yong Liu, Aiguo Tang

**Affiliations:** ^1^ Department of Laboratory Medicine, The Second Xiangya Hospital, Central South University, Changsha, Hunan 410011, China; ^2^ Department of Psychiatry, The Third Affiliated Hospital of Sun Yat-Sen University, Guangzhou, Guangdong 510631, China; ^3^ Department of Psychiatry, The Second Xiangya Hospital, Central South University, Changsha, Hunan 410011, China, Mental Health Institute of Central South University & Hunan Key Laboratory of Psychiatry and Mental Health, Changsha, China, China National Clinical Research Center on Mental Disorders (Xiangya) & China National Technology Institute on Mental Disorders, China; ^4^ Department of Biochemistry and Molecular Biology, Hunan University of Chinese Medicine, Changsha, Hunan 410208, China

**Keywords:** FES, OXT/OXTR, AVP/AVPR1a, CD38, mRNA

## Abstract

Schizophrenia (SZ) is a severe neuropsychiatric disorder with significant social cognition impairment. Increasing evidence has suggested that neuropeptides oxytocin (OXT) and arginine vasopressin (AVP) are important mediators of complex social cognition and behavior associates with SZ. In the present study, forty-three first-episode schizophrenia (FES) patients and forty-seven healthy controls (HC) were included. The peripheral mRNA expression of OXT, OXT receptor (OXTR), AVP, AVP 1a receptor (AVPR1a) and CD38 was determined by real-time quantitative polymerase chain reaction (RT-qPCR). The FES patients have a relatively higher mRNA level of OXT and OXTR genes and lower expression of AVP and CD38 genes than HC. No difference was found for AVPR1a between FES patients and HC. As for the sex difference, the mRNA expression of OXT and OXTR showed no difference in both male and female FES patients compared to HC group. The AVP and CD38 genes in female FES patients showed decreased mRNA expression than female HC. Our findings support disrupted OXT and AVP systems in the FES patients.

## INTRODUCTION

Schizophrenia (SZ) is a chronic debilitating neuropsychiatric disorder affecting almost 1% of the population worldwide [1]. Recently, the neuropeptide oxytocin (OXT) has attracted increasing attention in schizophrenia researches due to its roles in the improvement of social behavior of SZ patients [2, 3]. Nasal administration of OXT improved higher-order social cognition [4] and lower order facial emotion recognition [5] in SZ patients. In addition, the *Oxt*^*-/-*^ mice were more vulnerable to the prepulse inhibition (PPI) and presented an augmented effects of the N-methyl-D-aspartic acid (NMDA) receptor antagonist phencyclidine (PCP), suggesting that OXT has a therapeutic potential for SZ [6]. OXT is a nine-amino-acid neuropeptide and synthesized primarily in the neurons of the hypothalamic supraoptic nuclei (SON) and paraventricular nuclei (PVN). OXT receptor (OXTR) belongs to G-protein coupled receptor family with 7-transmembrane domains, and is thought to mediate the actions of OXT.

Recently, the variations of OXT and OXTR single nucleotide polymorphisms (SNPs) have been implicated with the risk for SZ and may contribute to symptom severity and treatment efficacy in SZ patients [7–9]. However, the endogenous OXT levels in SZ patients were still arguable, as it was reported higher [10], or lower in plasma [11] or no alteration in cerebrospinal fluid (CSF) [12] when compared with healthy controls (HC). These data suggested that a disrupted OXT system may closely associate with SZ. In addition, a lower OXTR mRNA level in the temporal cortex, a brain region highly implicated in social cognition, has been reported in the post-mortem of SZ patients [13]. This finding was consistent with significantly reduced OXT mRNA in the paraventricular nucleus of animal model [14]. CD38 is a transmembrane protein which regulates oxytocinergic neural transmission and OXT secretion (but not AVP secretion) in mice [15]. Higashida et al has found that OXT could be produced and packaged into vesicles in the hypothalamic neurons and posterior pituitary nerve endings in *Cd38*^*-/-*^ mice, but could not be released into the brain and bloodstream [16]. The *Cd38*^*-/-*^ mice presented abnormal social behaviors similar to those observed in *Oxt*^*-/-*^ and *Oxtr*^*-/-*^ mice, and supplementation of OXT and CD38 were able to restore the impaired social memory [15, 17].

Arginine vasopressin (AVP) is a neuropeptide homologous to OXT structurally and genetically [18] and shares the same mechanism of synthesis and release in the hypothalamus. AVP 1a receptor (AVPR1a) and AVP 1b receptor (AVPR1b) also belong to G protein-coupled membrane receptors and mediate actions of AVP in the brain. AVP is also involved in a range of social behaviors. Blocking the actions of AVP in the olfactory bulb impairs the social recognition abilities in rats [19], which can be manipulated by AVPR1a agonists and antagonists. The *AVP* gene is significantly associated with SZ [20] and AVP protein levels are decreased in the plasma of male schizophrenia patients [11]. Besides, AVP-deficient (Brattleboro) rats present impaired cognitive functioning [22] and intranasal administration of AVP analogue desmopressin can improve the symptoms (especially negative) of SZ [21]. In human patients, AVP is featured with increased vigilance and anxiety, whereas OXT is closely related with reduced anxiety [23].

It is noteworthy that the dosage of antipsychotic medication may influence OXT level [24] and blockage of dopamine receptors can elevate OXT concentration in plasma [25, 26]. However, it is unclear how psychiatric condition interacts with endogenous OXT in humans. In this study, we examined the relative mRNA expression of OXT, OXTR, AVP, AVPR1a and CD38 in HC and first-episode, unmedicated schizophrenia (FES) patients. Considering the actions of OXT and AVP are sexually dimorphic [27], we compared the gender-associated mRNA expression in HC and FES patients as well. We aim to determine whether our findings were consistent with previous literature and to provide evidence for the regulated OXT expression by CD38.

## RESULTS

**Figure 1:**
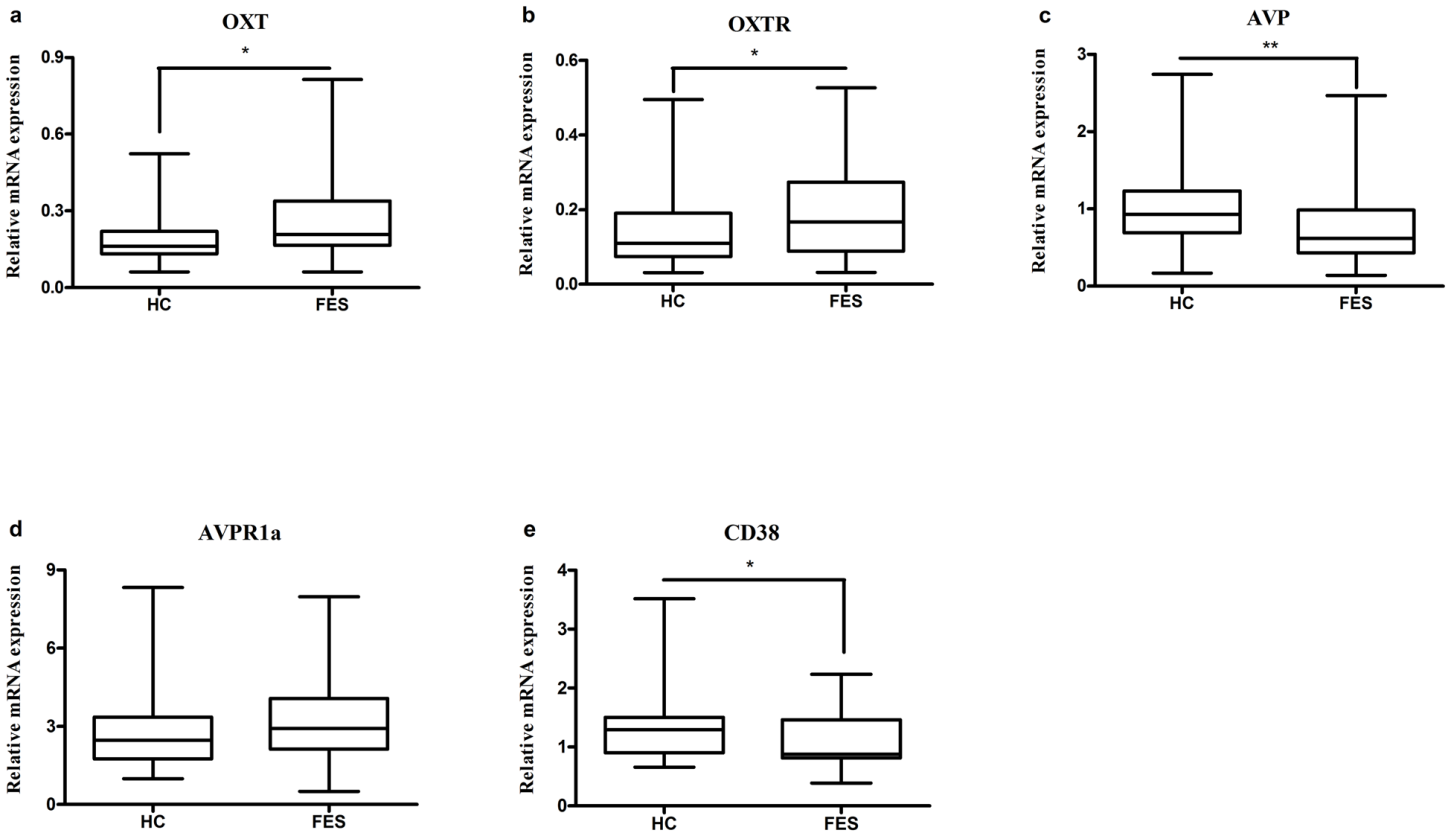
Representative of the relative mRNA expressions of OXT, OXTR, AVP, AVPR1a and CD38 genes * means *P* <0.05, ** means *P* <0.01, the horizontal line in the box represent median value, the crest line and bottom line of the box represent upper quartile and lower quartile, respectively. The unit of Y axis is arbitrary unit. **(a-c, e)**, the differences of HC with FES were presented; **(d)**, no significant difference was found between FES patients and HC.

The semi-quantitative measurement of mRNA expression of OXT, OXTR, AVP, AVPR1a, CD38 genes was performed using real-time PCR and β-actin was used as the internal control. The results were shown in Figure 1. Levels of OXT and OXTR mRNA were significantly higher (*P*=0.015 and *P*=0.037, respectively) in FES patients (0.2725 ± 0.1879 and 0.1945 ± 0.1229, respectively) than that in HC (0.1846 ± 0.0896 and 0.1443 ± 0.0156, respectively). The expression of AVP and CD38 mRNA was significantly decreased (*P*=0.001 and *P*=0.012, respectively) in FES patients (0.7359 ± 0.4639 and 1.0861 ± 0.4564, respectively) compared with those in HC (1.0378 ± 0.4905 and 1.3830 ± 0.6691, respectively). There was no difference (*P*=0.300) for the levels of AVPR1a mRNA between FES patients (3.1512 ± 1.6290) and HC (3.0950 ± 2.0266).

**Figure 2:**
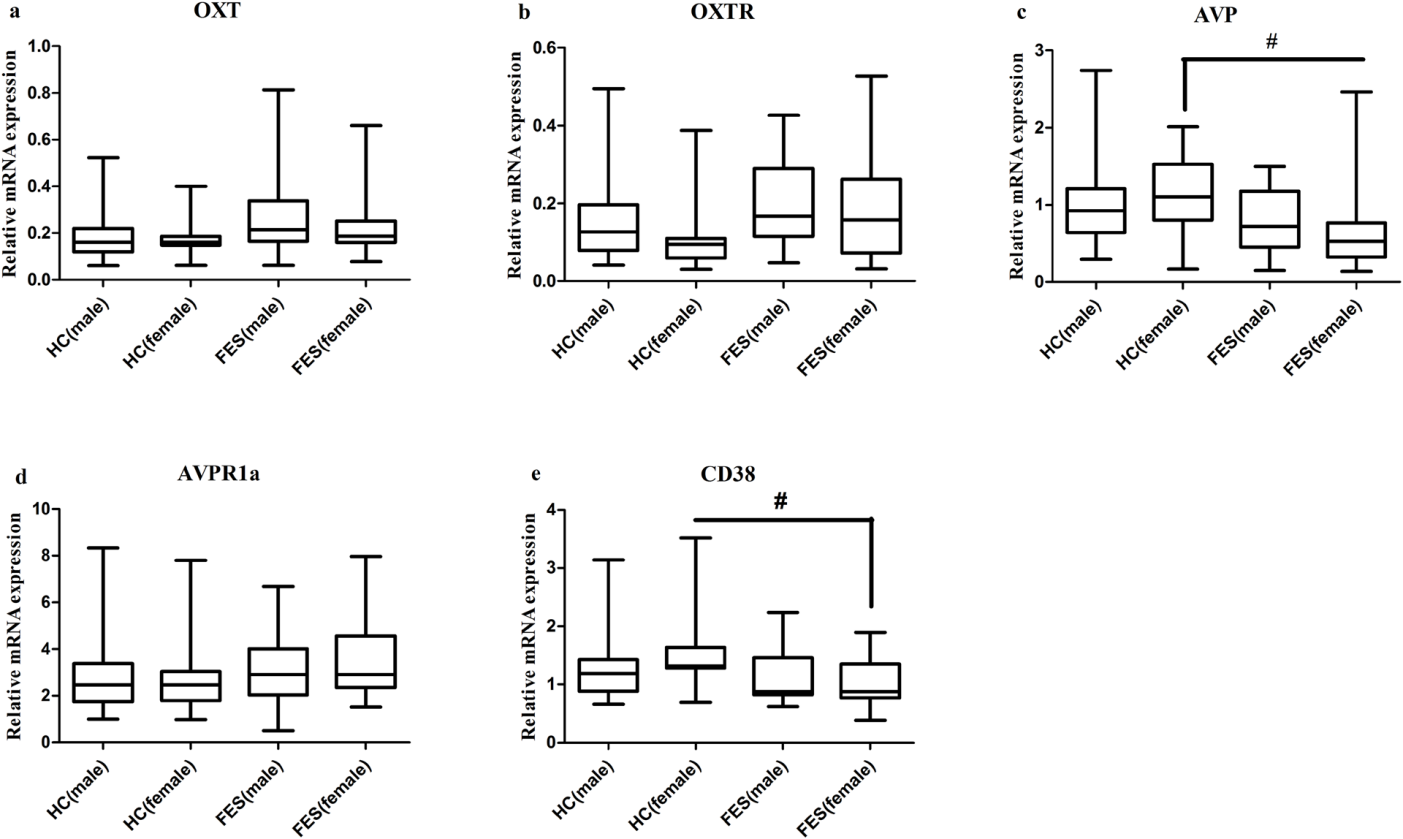
Representative of the relative mRNA expressions in each gender of OXT, OXTR, AVP, AVPR1a and CD38 genes # means *P* <0.0125, the horizontal line in the box represent median value, the crest line and bottom line of the box represent upper quartile and lower quartile, respectively. The unit of Y axis is arbitrary unit. **(c, e)**, the differences of female HC with female FES were presented; **(a, b, d)**, no significant differences were noted in these variables either from a cross-sex comparison within FES group or HC group, or from a same-sex comparison between the above two groups.

The sex difference of these five genes was presented in Figure 2. The intra-group comparisons of mRNA expression between males and females showed no difference both in FES patients and HC group. The mRNA expression of OXT and OXTR both in male and female FES patients (male: 0.2861 ± 0.1985 and 0.2027 ± 0.1139, female: 0.2496 ± 0.1724 and 0.1813 ± 0.1399, respectively) showed no difference compared with HC group (male: 0.1845 ± 0.0935 and 0.1554 ± 0.1079, female:0.1846 ± 0.0855 and 0.1264 ± 0.1062, respectively). Female FES patients (0.6571 ± 0.5457 and 1.0331 ± 0.4190, respectively) showed significantly decreased (*P*=0.003 and *P*=0.002, respectively) AVP and CD38 mRNA than female HC (1.1096 ± 0.5325 and 1.6094 ± 0.8156, respectively).

## DISCUSSION

Although antipsychotic medication can influence OXT level [24], it is not clear how psychiatric conditions interact with endogenous OXT in humans. In the present study, we provide evidence that the OXT and OXTR mRNA expression in peripheral blood lymphocytes of HC was significantly lower than FES patients. In addition, the AVP and CD38 mRNA were significantly decreased in FES patients, but the AVPR1a mRNA showed no difference between two groups.

Previously, most of the studies suggested that plasma OXT level in SZ patients was significantly lower [11, 28, 29], although some argued an increased or unchanged OXT level in CSF [30]. A higher endogenous OXT level boosted total recognition (four discrete emotions: happiness, sadness, anger and neutral) in both SZ patients and HC [31] and plasma OXT level was used to predict the ability to correctly identify facial emotion in SZ patients [28]. Intranasal administration of 24 IU of OXT in healthy humans increased the concentration of OXT in CSF and blood [32]. In clinical experiments, SZ patients who received 3 weeks of 20 or 40 IU OXT twice a day presented reduced scores on the Positive and Negative Symptom Scale (PANSS) and significant improvement of clinical symptoms [33–35]. In our study, we found that the mRNA expression of OXT and OXTR in the peripheral blood lymphocytes were significantly higher in FES patients, especially the male FES patients were slightly higher, and there is no gender difference both in HC and FES group. This was consistent with higher plasma OXT levels in SZ patients than HC reported in several studies [10, 31], although Jobst A et al. reported decreased plasma OXT level in male SZ patients [11]. As for the gender differences, males, not females, showed slight increase of OXT and OXTR mRNA expression. This is in agreement with the positive effects of estrogen for the treatment of SZ patients [37] and the concept that males are more vulnerable than females to develop schizophrenia, with an incidence risk ratio of 1.4 [36]. So, we speculated that the dysfunctional production, transportation or release of OXT in the brain may lead to abnormal peripheral expression, although the exact mechanism is not clear. The up-regulated mRNA expression of OXT and OXTR in peripheral blood lymphocytes may be the compensatory outcomes of OXT system dysfunction in the brain.

AVP is an antidiuretic hormone which also plays an important role in social behavioral modulation. We found that AVP mRNA was significantly lower in FES patients, particularly in females. Besides, the AVP level was reported typically higher in males than females in a recent review [38] and Sacher et al. has found the sex-dependent associations between cognition and emotion brain networks through neuroimaging observation [39]. So, these difference may be explained by that female FES patients are more vulnerable to the disrupted AVP system. This result was in accordance with two recent studies that peripheral AVP levels in SZ patients were lower than that in HC [11, 40]. In contrast, Rubin and her colleagues demonstrated that an increased serum AVP levels compared to HC associated with greater severity of positive symptoms in female SZ patients [41]. Although the association between negative symptoms and AVP levels still remains unclear, the AVP-deficient rats (Brattleboro) displayed explicit features of SZ [42] and intranasal administration of AVP analogue can greatly improve negative symptoms [21]. Therefore, AVP has been proposed as an promising substitution or adjunct of antipsychotic drugs for SZ patients. and In terms of the inconsistent literature reports, we speculated that higher levels of AVP with greater positive symptoms were related to the dysregulated biological stress response system [43], while the lower AVP levels accompanied with negative symptom as shown in this study. Although AVPR1a is most abundantly expressed in the brain and previous studies have demonstrated that AVPR1a is implicated in social behavior in animals and human [44], the expression of AVPR1a mRNA in FES patients was approximately equal to HC in this study, implying a normal function of receptor system potentially.

CD38 was originally examined extensively in the field of chronic leukocyte leukemia malignancy and is a marker of HIV infection in blood cells. *CD38* gene expression in peripheral cells is positively correlated with plasma OXT levels [45], suggesting that CD38 facilitates OXT release. Interestingly, the CD38 null mutation has no effect on AVP release [15] and the underpinning mechanism was not clear. As previously reported, significantly lower plasma and CSF concentration of OXT in CD38^-/-^ mice could be reversed by the CD38 re-expression [15], which means that the dysfunction of CD38 could lead to the abnormality of OXT-OXTR system. In this study, we detected overt difference of CD38 gene expression in peripheral blood lymphocytes between FES patients and HC. The basic mRNA expression of CD38 gene was significantly different between males and females in HC and the mRNA expression in female FES patients were lower than that in female HC. Therefore, the expression difference in males and females should be treated discriminatively. We speculate that the significantly decreased CD38 expression may cause the up-regulation of OXT and OXTR genes in a compensatory mechanism to maintain the normal function of the CD38-OXT-OXTR pathway.

The SZ patients recruited in this study met with the drug-free criteria strictly. This is because it is not clear whether AVP and its receptors are modulated by antipsychotic medication. Therefore, the drug-free patients would be the best subjects to rule out the potential interference of drugs.

It has to be noted that we did not group the subjects by ages and therefore the influence of disease course was not taken into account. A further independent study with larger sample size is indeed more proper. Whether the inter/intra-group difference in some gene expression are correlated to the estrogen or/and other hormones should also be illuminated. In addition, this study examined the peripheral blood samples instead of the brain, which limits the generalizability of the current study.

## MATERIALS AND METHODS

### Subjects

Fourty-three patients were recruited (Table 1) for mRNA experiment from the Department of Psychiatry in the Second Xiangya Hospital, Central South University. They were diagnosed with SZ by two senior psychiatrists according to the criteria of the Diagnostic and Statistical Manual of Mental Disorders, Fifth Edition (DSM-V). All of the patients were not treated with any antipsychotic drug and signed an informed consent before participating in this study. Patients with comorbid mental disorders, a history of traumatic brain injury or intellectual disability, or serious somatic diseases, or active or chronic inflammatory or autoimmune diseases were excluded. This study was approved by the Ethics Committee of the Second Xiangya Hospital, Central South University.

**Table 1:** Demographic data for FES patients and HC

	FES	HC	*P*-value
Sex	N(%)	N(%)	
Male	27(62.8)	29(61.7)	0.915
Female	16(37.2)	18(38.3)	
Total	43	47	
Age(Mean±SD)	22.26±4.49	23.32±2.68	0.06
Male	21.70±3.24	22.79±2.73	
Female	23.19±5.73	24.11±2.47	
PANSS			
Total	72.57±20.50	/	
Positive	19.79±4.97	/	
Negative	19.33±6.85	/	
General	33.45±9.77	/	

### Sample collection

10 ml peripheral venous blood samples were collected from fasting FES patients and HC in the morning into an anticoagulant tube by the well-trained nurses. Samples with coagulation and hemolysis were discarded and repeated freezing-thawing was avoided. Peripheral blood mononuclear cells (PBMCs) were isolated and stored at -80°C until the RNA was extracted.

### RT-qPCR

#### RNA isolation

The RNA was extracted from lymphocytes in MagNA Pure LC2.0 Automatic extractor with MagNA Pure LC Total Nucleic Acid Isolation Kit–High Performance: automatic RNA extraction using magnetic beads (Roche Diagnostics, IN, USA). The isolation protocol was performed according to the manufacturer's instructions. After extraction, RNA was quantified with a NanoDrop2000 (Thermo fisher Scientific, USA); Purity ranging between 1.8 and 2.1 was deemed as a good RNA quality.

#### cDNA synthesis

After completion of the previous step, the complementary DNA (cDNA) was produced using the Transcriptor First Strand cDNA Synthesis Kit (Roche Applied Science).

### Primer design

The primer sequences of all target genes were referred from references (OXT/OXTR/AVPR1a) or designed (AVP/CD38) by Primer Premier (version 5.0) according to the mRNA sequences found in the NCBI at http://www.ncbi.nlm.nih.gov. Sequences and size of the primer pairs are shown in Table 2.

**Table 2:** Primers sequences of the target genes and reference gene

Gene	Primer sequences	Amplicon length(bp)
OXT	F-GCTGAAACTTGATGGCTCCG	67
	R-TTCTGGGGTGGCTATGGG	
OXTR	F-CTGAACATCCCGAGGAACTG	84
	R-CTCTGAGCCACTGCAAATGA	
AVP	F-ACCTGGAGCTGAGACAGTGCCT	231
	R-TCACGCAGCTCTCGTCGTT	
AVPR1a	F-CTTTTGTGATCGTGACGGCTTA	114
	R-TGATGGTAGGGTTTTCCGATTC	
CD38	F-CCTGAGATGAGACATGTAGACTGC	160
	R-TTCTGCTCCAAAGAAGAATCTTGTTG	
β-actin	F-TCCCTGGAGAAGAGCTACGA	136
	R-TGAAGGTAGTTTCGTGGATGC	

### Quantitative PCR

The qPCR reaction contained 10 μl 2×SYBR Green Mastermix (Roche Applied Science), 1μl of each primer pair (5μM) and 5μl of template cDNA in a 20 μl reaction volume. All reactions were completed with the Roche LightCycler 480 (Roche) using the LightCycler 480 SYBR Green I Master (Roche) with the following cycling conditions: 95°C for 10 minutes, followed by 40 cycles of denaturation at 95°C for 10 seconds, annealing at 60°C for 10 seconds and extension at 72°C for 20 seconds. A single fluorescence read was taken at the end of each 72°C step. The specificity of the amplification was controlled by melting curve analysis. Experiments were performed in duplicate for each sample. The mean value of the replication for each sample was calculated and presented as a cycle threshold (Ct). Expression of the target genes as the fold increase or decrease was relative to the expression of β-actin. The relative mRNA levels of target genes were determined as 2^−ΔΔCt^.

### Statistical analysis

Statistical analyses were performed with the SPSS (version 18.0; Chicago, IL, USA). Data were presented as mean±standard deviation (SD) for normal distribution variables (at Kolmogorov-Smirnov test) and median (quartile range) for non-normal distribution variables. Continuous variables were compared using Student’s t-test or the Mann-Whitney U-test as appropriate. Comparison between the two groups were considered statistically significant if *P*<0.05, Bonferroni correction was used to adjust our results for multiple comparisons (0.05/4 = 0.0125).

## CONCLUSION

We have shown that the mRNA levels of OXT and OXTR in FES patients are relatively higher than that in HC, and the expression of AVP and CD38 was lower than HC. While the mRNA expression of AVPR1a gene showed no difference between the two groups, indicating a dysfunctional OXT system in the FES patients. The relationship between gender difference and gene expression should take into consideration as well. At present, the treatment and therapy choices for SZ patients are very limited and we hope this study will establish the potentially useful biomarkers and drug-targets for both the diagnosis and treatment of SZ patients in future.

## Abbreviations

FES: first-episode schizophrenia
OXT: oxytocin
OXTR: oxytocin receptor
AVP: arginine vasopressin
AVPR1a: AVP 1a receptor
AVPR1b: AVP 1b receptor
SZ: schizophrenia
HC: healthy controls
RT-qPCR: real-time quantitative polymerase chain reaction
SON: supraoptic nuclei
PVN: paraventricular nuclei
SNPs: single nucleotide polymorphisms
CSF: cerebrospinal fluid
DSM-V: Diagnostic and Statistical Manual of Mental Disorders, Fifth Edition
SD: standard deviation
PANSS: Positive and Negative Symptom Scale
